# Interaction between synaptic dynamics and synaptic configuration determines the phase of the response to rhythmic inputs

**DOI:** 10.1186/1471-2202-12-S1-P128

**Published:** 2011-07-18

**Authors:** Bruce P Graham

**Affiliations:** 1 Computing Science and Mathematics, School of Natural Sciences, University of Stirling, Stirling, FK9 4LA, UK

## 

The postsynaptic response of a neuron to time-varying inputs is determined by the interaction of presynaptic spike times with the short-term dynamics of each synapse. For a neuron driven by stochastic synapses and operating in afluctuation-driven regime, synaptic depression results in a quite different postsynaptic response to a large population input depending on how correlated in time the spikes across individual synapses are [1,2]. Here we show that not only the rate but the phase of the postsynaptic response to a rhythmic population input varies as a function of synaptic dynamics and synaptic configuration, which contributes to input correlations.

In computer simulations, a single- compartment spiking model neuron is fed inputs via a synaptic pathway containing M=512 vesicle release sites. The pathway is configured in different ways by assigning release sites equally between varying numbers of active zones (N). Each active zone is driven by a particular input spike train. Spike trains to different active zones have the same time-varying mean frequency but individual spikes are uncorrelated in time. A completely uncorrelated pathway consists of 512 active zones containing a single release site, each driven by an independent presynaptic spike train. In contrast, a completely correlated pathway consists of a single active zone containing 512 releasable vesicles that may all release independently on the arrival of a spike from a single presynaptic cell. Each release site is modelled stochastically: a site may or may not contain a vesicle; an empty release site may be refilled at a fixed rate; a full site may release its vesicle with a given probability on arrival of a presynaptic spike. The refilling rate may be slow compared to the arrival rate of spikes, resulting in synaptic depression. The probability of vesicle release may increase with each spike, decaying back to baseline at a slow rate, resulting in facilitation of release. In the results shown here, the refilling rate is 2/s and the facilitation decay rate is also 2/s. The input signal is a 30Hz carrier frequency that is modulated sinusoidally between 50Hz and 10Hz at different modulation frequencies. This signal is carried by up to 512 independent non-homogeneous Poisson-distributed spike trains.

**Figure 1:**
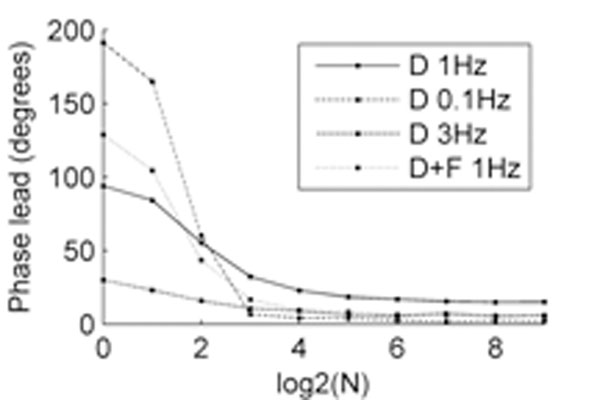
Phase of response to rhythmic population inputs for different synaptic configurations and modulation frequencies. D: depression; D+F: depression + facilitation.

The phase of the output spiking response (obtained from multiple runs with the same and different input trains), relative to the input signal, is plotted against the number of active zones (N) in Figure 1. At all modulation frequencies (0.1, 1, 3Hz) and in all configurations, synaptic depression results in the output response leading the input oscillations. The lead increases as the inputs become more correlated (decreasing N). The effect is amplified by facilitation for correlated synapses (N=1 or 2), but reduced for less correlated pathways. Also, the lead decreases with increasing modulation frequency for correlated synapses, but is maximal at 1Hz for uncorrelated pathways.
